# ZupT Facilitates Clostridioides difficile Resistance to Host-Mediated Nutritional Immunity

**DOI:** 10.1128/mSphere.00061-20

**Published:** 2020-03-11

**Authors:** Joseph P. Zackular, Reece J. Knippel, Christopher A. Lopez, William N. Beavers, C. Noel Maxwell, Walter J. Chazin, Eric P. Skaar

**Affiliations:** a Department of Pathology and Laboratory Medicine, Perelman School of Medicine, University of Pennsylvania, Philadelphia, Pennsylvania, USA; b Division of Protective Immunity, Children’s Hospital of Philadelphia, Philadelphia, Pennsylvania, USA; c Institute for Immunology, Perelman School of Medicine, University of Pennsylvania, Philadelphia, Pennsylvania, USA; d Department of Pathology, Microbiology, and Immunology, Vanderbilt University Medical Center, Nashville, Tennessee, USA; e Vanderbilt Institute for Infection, Immunology, and Inflammation, Vanderbilt University Medical Center, Nashville, Tennessee, USA; f Department of Biochemistry, Center for Structural Biology, Vanderbilt University, Nashville, Tennessee, USA; g Department of Chemistry, Center for Structural Biology, Vanderbilt University, Nashville, Tennessee, USA; University of Michigan-Ann Arbor

**Keywords:** *Clostridium difficile*, host-pathogen interactions, infectious disease, nutrient transport

## Abstract

Clostridioides difficile is a spore-forming bacterium that causes severe colitis and is a major public health threat. During infection, C. difficile toxin production results in damage to the epithelium and a hyperinflammatory response. The immune response to CDI leads to robust neutrophil infiltration at the sight of infection and the deployment of numerous antimicrobials. One of the most abundant host immune factors associated with CDI is calprotectin, a metal-chelating protein with potent antimicrobial activity. Calprotectin is essential to the innate immune response to C. difficile and increasing levels of calprotectin correlate with disease severity in both adults and children with CDI. The fact that C. difficile persists in the presence of high levels of calprotectin suggests that this organism may deploy strategies to compete with this potent antimicrobial factor for essential nutrient metals during infection. In this report, we demonstrate that a putative zinc (Zn) transporter, ZupT, is employed by C. difficile to survive calprotectin-mediated metal limitation. ZupT is highly expressed in the presence of calprotectin and is required to protect C. difficile against calprotectin-dependent growth inhibition. When competing against wild-type C. difficile, *zupT* mutants show a defect in colonization and persistence in a murine model of infection. Together these data demonstrate that C. difficile utilizes a metal import system to combat nutritional immunity during CDI and suggest that strategies targeting nutrient acquisition in C. difficile may have therapeutic potential.

**IMPORTANCE** During infection, pathogenic organisms must acquire essential transition metals from the host environment. Through the process of nutritional immunity, the host employs numerous strategies to restrict these key nutrients from invading pathogens. In this study, we describe a mechanism by which the important human pathogen Clostridioides difficile resists transition-metal limitation by the host. We report that C. difficile utilizes a zinc transporter, ZupT, to compete with the host protein calprotectin for nutrient zinc. Inactivation of this transporter in C. difficile renders this important pathogen sensitive to host-mediated metal restriction and confers a fitness disadvantage during infection. Our study demonstrates that targeting nutrient metal transport proteins in C. difficile is a potential avenue for therapeutic development.

## Abstract (precis)

During infection, pathogenic organisms must acquire essential transition metals from the host environment. Through the process of nutritional immunity, the host employs numerous strategies to restrict these key nutrients from invading pathogens. In this study, we describe a mechanism by which the important human pathogen Clostridioides difficile resists transition-metal limitation by the host. We report that C. difficile utilizes a zinc transporter, ZupT, to compete with the host protein calprotectin for nutrient zinc. Inactivation of this transporter in C. difficile renders this important pathogen sensitive to host-mediated metal restriction and confers a fitness disadvantage during infection. Our study demonstrates that targeting nutrient metal transport proteins in C. difficile is a potential avenue for therapeutic development.

## INTRODUCTION

Transition metals, including iron (Fe), manganese (Mn), and zinc (Zn), are essential micronutrients for all living organisms. These metals are thus key resources in the battle between a host and pathogen during infection. Upon colonization of the mammalian host, pathogens must acquire transition metals from complex, diverse, and largely metal-restricted environments (1, 2). Pathogens employ numerous strategies to scavenge metals from the host, including pirating metal from host proteins, releasing metal-chelating metallophores, and expressing high-affinity metal import systems (2). To combat this, vertebrates exploit the pathogen requirement for transition metals by producing factors that deplete the environment of free transition metals in a process termed nutritional immunity (3, 4). One such factor is calprotectin, a heterodimer of S100A8 and S100A9 proteins. Calprotectin is abundant in the cytoplasm of neutrophils and chelates multiple transition metals, including Fe, Mn, and Zn (5). Calprotectin-mediated metal limitation is antimicrobial to numerous pathogens and is critical to combating infection (6–9). Defining the battle for transition metals between host and pathogen is important to understanding the factors that impact the outcome of infection, and has the potential to uncover novel therapeutic strategies for targeting pathogens.

The spore-forming bacterium Clostridioides difficile is the most commonly reported health care-associated pathogen in the United States and a global public health threat (10). The primary risk factor for C. difficile infection (CDI) is antibiotic treatment, which disturbs the resident microbial community in the gastrointestinal tract and reduces colonization resistance against the pathogen (11). Notably, the first-line therapy for C. difficile is also antibiotic treatment, which further perturbs the gut microbiota and increases risk for recurrent infection. Over the past decade, the rate, severity, and economic cost of CDI have risen dramatically in both children and adults (10, 12). This highlights the urgent need for new antibiotic targets and novel therapeutic strategies for treating CDI.

Recent work from our group and others has demonstrated that fecal calprotectin is associated with CDI in humans and high levels of calprotectin are correlated with increased disease severity (7, 13–15). We have further demonstrated that calprotectin is antimicrobial against C. difficile and calprotectin-mediated metal limitation is an essential host immune response during CDI (7). Despite calprotectin’s antimicrobial properties and high concentrations in the gastrointestinal tract during severe infections, C. difficile persists in this metal-limited environment. This suggests that C. difficile employs strategies to compete with the host for essential transition metals during infection, allowing for persistence. The mechanisms by which C. difficile combats host nutritional immunity have not been explored and may represent a new therapeutic target for treatment of CDI.

In this study, we investigated the role for a putative Zn transporter in C. difficile during host-mediated metal limitation. ZupT homologs in Salmonella enterica and Escherichia coli are important for metal scavenging in these organisms, but the role for ZupT in C. difficile has not been experimentally explored (16–18). We demonstrate that *zupT* is highly upregulated in the presence of recombinant calprotectin and is required for survival of calprotectin-mediated metal limitation *in vitro*. Furthermore, we show that ZupT imports Zn when C. difficile is metal starved and ZupT-deficient strains of C. difficile are less fit in a mouse model of infection. Together these results show that ZupT is an important factor used by C. difficile to combat host nutritional immunity during CDI.

## RESULTS

### C. difficile upregulates the putative Zn transporter ZupT during host-mediated metal limitation.

**Fig. 1:**
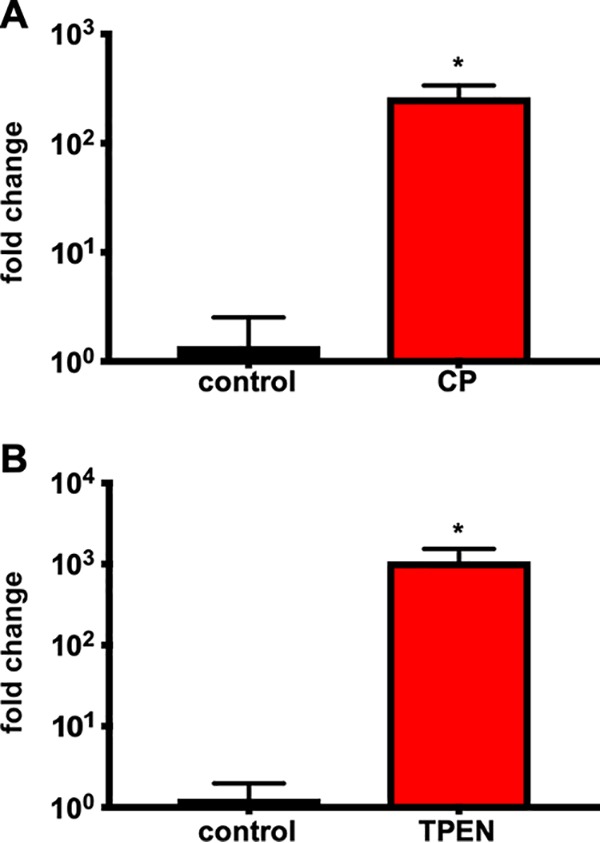
*zupT* is upregulated during nutrient metal limitation. C. difficile was grown in the presence of 0.35 mg/ml calprotectin (A) or 50 μM TPEN (B). *zupT* transcripts were measured via qRT-PCR and are shown as fold change from untreated cells (*n* = 3) with standard deviation. CP, calprotectin. Statistical significance was determined by *t* test (*, *P < *0.05).

Calprotectin is essential to the immune response to CDI and calprotectin-mediated metal limitation is antimicrobial to C. difficile (3, 7). An RNA sequencing study by our group showed that the putative Zn transporter ZupT is one of the most highly upregulated genes in the presence of calprotectin *in vitro* (19). To begin to assess the contribution of ZupT to the response to host-mediated metal starvation, the transcriptional induction of *zupT* during treatment with recombinant calprotectin was validated using reverse transcription-quantitative PCR (qRT-PCR). In the presence of 0.35 mg/ml calprotectin, a >200-fold transcriptional increase of *zupT* was observed compared to the untreated controls (Fig. 1A). To confirm that this response was specific to metal limitation, C. difficile was treated with a chemical chelator *N*,*N*,*N’*,*N’-*tetrakis (2-pyridinylmethyl)-1,2-ethanediamine (TPEN; 50 μM). Exposure to TPEN-mediated metal starvation revealed a 500-fold increase in *zupT* transcripts compared to the untreated control (Fig. 1B). These results demonstrate that transcription of *zupT* is a robust response by C. difficile to metal limitation.

### Inactivation of *zupT* decreases resistance to metal limitation.

**FIG 2:**
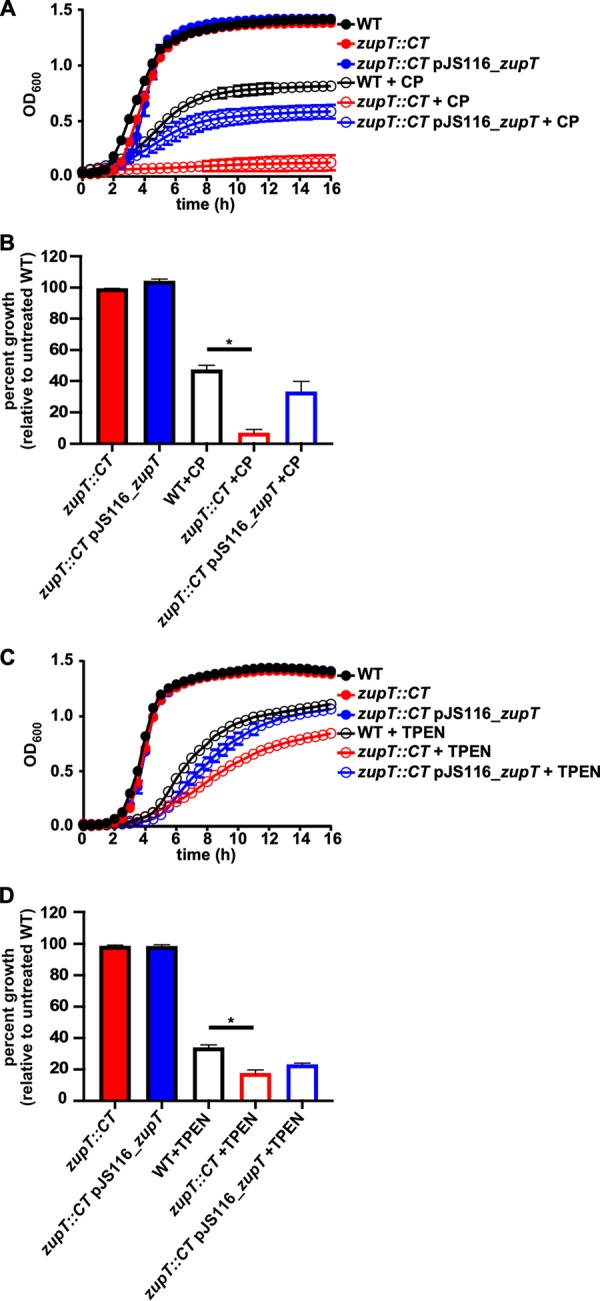
ZupT is required for growth during metal limitation. Growth curves of WT C. difficile, mutant *zupT*::CT, and complemented *zupT*::CT pJS116_*zupT* grown in the presence of 0.35 mg/ml calprotectin (A) or 50 μM TPEN (C). Percent growth based off maximum OD at 6 h is shown for calprotectin (B) and TPEN (D). Statistical significance was determined by *t* test (*, *P < *0.05).

Based on the increased expression of *zupT* in the presence of calprotectin, we hypothesized that the encoded protein may play a central role in Zn acquisition during metal limitation by the host. To test this, a strain of C. difficile inactivated for *zupT* (*zupT*::*CT*) was generated using the ClosTron system (20). The lack of *zupT* rendered the bacteria more sensitive to calprotectin-mediated metal starvation, as growth was delayed over time in the presence of 0.35 mg/ml calprotectin and overall percent growth was reduced by 80% compared to the wild-type (WT) strain at 6 h (Fig. 2A and B). Similar results were observed when the growth of the bacteria was assessed in 50 μM TPEN, which led to a 40% reduction in growth of the mutant compared to the WT strain (Fig. 2C and D). The growth of *zupT*::*CT* in either condition is recovered by expressing *zupT* in *trans* under the control of the predicted promoter-containing region upstream of *zupT* (*ZupT*::*CT* pJS116_*zupT*; Fig. 2A to D). Taken together, these data suggest that ZupT functions to obtain metals, such as Zn, in the face of host nutritional immunity and may play an important role in reducing the effect on C. difficile of nutrient metal starvation.

### ZupT imports Zn during metal limitation.

**FIG 3:**
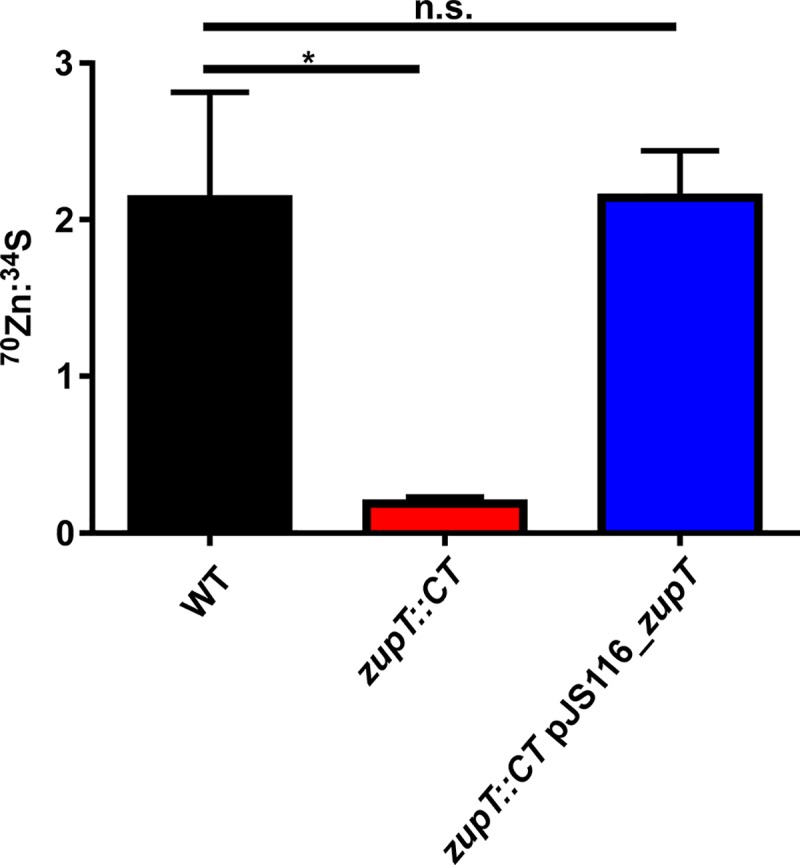
ZupT imports Zn in response to metal limitation. ^70^Zn uptake measured by ICP-MS. WT C. difficile, mutant *zupT*::CT, and complemented *zupT*::CT pJS116_*zupT* were pregrown in the presence of 50 μM TPEN and then supplemented with ^70^Zn. Following incubation, ^70^Zn uptake was measured. Statistical significance was determined by *t* test (*, *P < *0.05; n.s., not significant).

ZupT is highly expressed during competition with calprotectin and zinc uptake is essential to C. difficile survival during calprotectin-mediated metal starvation; however, it remains unknown if this putative metal transporter imports Zn into the cell (7, 19). To investigate the ability of ZupT to take up Zn, cell cultures were grown in Zn-limited media and pulsed with a minor Zn stable isotope (^70^Zn) prior to analysis of intracellular ^70^Zn levels by inductively coupled plasma mass spectrometry (ICP-MS). The *zupT*::*CT* strain displayed a 10-fold reduction in intracellular ^70^Zn compared to the WT strain in these experiments (Fig. 3). The uptake of ^70^Zn was restored in the *zupT*::*CT* pJS116_*pzupT-zupT* complemented strain. These data demonstrate the direct contribution of ZupT to increasing intracellular Zn concentrations as a strategy to combat Zn-limiting conditions (Fig. 3), and strongly support the assignment of ZupT as a Zn transporter.

### ZupT mutants show a colonization defect in the gastrointestinal tract during infection.

**FIG 4:**
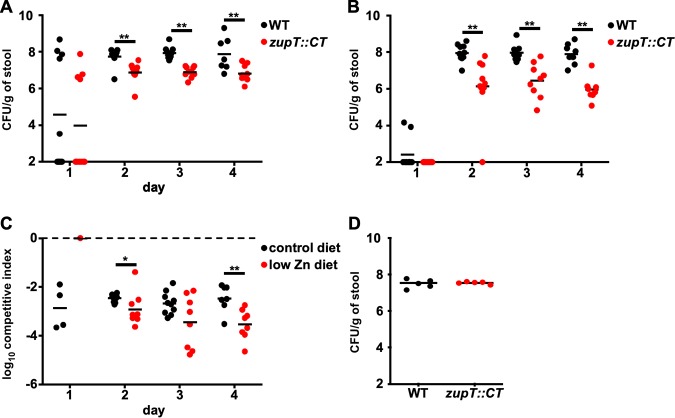
Strains of C. difficile inactivated for *zupT* exhibit a defect in a competitive murine model of C. difficile infection. WT C. difficile and *zupT*::CT spores were orally gavaged at a 1:1 ratio into antibiotic pretreated mice. Mice were fed a low-Zn diet (A) or a control diet (B) prior to infection and CFU were measured for each strain using differential media. Competitive index (*zupT*::CT/WT) was measured for each dietary condition (C). Each individual point is representative of an individual mouse and the bar represents the mean. CFU were enumerated during a 4-day time course of infection of mice on the control diet infected with WT or *zupT*::*CT* spores (D). Statistical significance was determined by *t* test (*, *P < *0.05; **, *P < *0.01).

The reduced ability of *zupT*::*CT* to resist Zn starvation in the presence of the innate immune protein calprotectin suggests that ZupT may be required in a mouse model of CDI. To test this, mice were placed on a custom formulated diet that is Zn-free (7). This Zn-free diet was supplemented with standard levels of Zn (control diet: 29 mg/kg) and animals were fed the diet for 1 week prior to infection (7). Mice were then coinfected with WT and *zupT*::*CT* spores of C. difficile at a 1:1 ratio and disease was monitored for 4 days. Following treatment with the control diet, the WT strain colonized to ∼10^8^ CFU per gram of stool (Fig. 4A). The *zupT*::*CT* strain displayed a 1 log reduction in colonization compared to the WT strain (Fig. 4A). Next, to examine the role of ZupT during conditions of Zn deficiency, we coinfected mice that were fed a custom formulated diet lacking Zn supplementation (low-Zn diet: 0 mg/kg). Following infection, we observed a 2 to 3 log defect in the *zupT*::CT strain under these extreme Zn-limited conditions (Fig. 4B). In both dietary conditions, the *zupT*::CT mutant showed a significant reduction in competitive index compared to WT and this reduction was increased in the low Zn diet (Fig. 4C). To ensure the *zupT*::*CT* strain does not contain an innate colonization defect, mice fed the control diet were mono-infected with either WT or *zupT*::*CT* strains. There were no signification differences in colonization between the WT or *zupT*::*CT* strain throughout the infection (Fig. 4D). Together, these data suggest that ZupT is required for C. difficile to overcome nutrient Zn limitation imposed by the host during CDI.

## DISCUSSION

Transition metals are essential micronutrients for all living organisms, and invading pathogens must acquire these metals in the host environment to colonize and establish infection (2). This is complicated even further in the gastrointestinal tract, as enteric pathogens must also compete with resident microbiota for transition metals. Calprotectin-dependent nutritional immunity is essential to defend against CDI (7). However, recent studies have demonstrated that increased fecal calprotectin is a hallmark of severe CDI, suggesting that during severe infection, C. difficile must employ strategies to persist and thrive in the gastrointestinal tract despite nutritional immunity (7, 13–15). In this study, we explored a potential mechanism by which C. difficile competes with the host for transition metals during infection. Previously, our group demonstrated that calprotectin is an essential component of the innate immune response to CDI and calprotectin-mediated transition metal limitation is inhibitory to C. difficile growth (7). Furthermore, we showed that subinhibitory concentrations of calprotectin dramatically alter the transcriptional profile and metabolism of C. difficile *in vitro* (19).

Here, we explored the role of one of the most highly upregulated genes in the presence of calprotectin, *zupT*. Previous studies have shown that *zupT* is upregulated under metal stress, but this putative metal transporter has not been studied experimentally in C. difficile (19, 21, 22). We demonstrated that *zupT* expression was robustly induced in the presence of calprotectin or a chemical chelator, and that inactivation of *zupT* decreased the ability of C. difficile to grow in the presence of subinhibitory concentrations of calprotectin. Using ICP-MS, we further demonstrated that *zupT* mutant cells have a defect in heavy Zn uptake, suggesting that ZupT is indeed a bona fide Zn transporter in C. difficile. In mice fed standard Zn diets, as well as Zn-free diets, strains of C. difficile lacking functional ZupT showed decreased fitness compared to wild type. Taken together, this suggests that C. difficile employs strategies to compete with the host and the resident microbiota for metals during infection.

ZupT has been reported in several studies to be one of the most highly upregulated genes in C. difficile during metal stress, suggesting this importer plays a key role in metal homeostasis for this important pathogen (19, 21, 22). However, despite this strong transcriptional response, inactivation of *zupT* does not completely inhibit the ability of C. difficile to colonize the host or grow in the presence of lower concentrations of calprotectin (Fig. 2 and 4). This suggests that C. difficile harbors additional metal import systems that may play similar roles in acquisition of Zn during. In support of this hypothesis, multiple putative Fe (CDR20291_1545-1548, CDR20291_1326-1328, CDR20291_2771-2774) and cation (CDR20291_0516-0157) transporters are transcriptionally increased in the presence of calprotectin (19). The phenomenon of functional redundancy in metal transport is shared among numerous enteric pathogens, including Enterococcus faecalis, E. coli, and multiple strains of *Salmonella* (16, 23–25). This has been specifically reported for ZupT homologs in several organisms. For example, the loss of ZupT in combination with the ZnuABC transporter in uropathogenic E. coli leads to a cumulative effect on fitness during urinary tract infections, whereas there was no decrease observed in a strain lacking only ZupT (24). In Salmonella enterica infection, strains lacking ZupT are attenuated in natural resistance-associated macrophage protein 1 (Nramp1)^+/+^ mice, but strains lacking both ZnuABC and ZupT exhibit markedly pronounced attenuation during infection (16). Future studies that simultaneously interrogate multiple Zn-transport systems are needed to define the mechanisms of Zn import in C. difficile during host nutritional immunity.

Taken together, these data demonstrate that metal import in C. difficile is an important response to host-mediated nutritional immunity during infection. Future work is needed to characterize the response of C. difficile to transition metal limitation in the gut and to define the role for functional redundancy in metal transporters, as these pathways represent an intriguing target for therapeutic development.

## MATERIALS AND METHODS

### Ethics statement.

All animal experiments under protocol M1700053 were reviewed and approved by the Institutional Animal Care and Use Committee of Vanderbilt University. Procedures were performed according to the institutional policies, NIH guidelines, Animal Welfare Act, and American Veterinary Medical Association guidelines on euthanasia.

### Bacterial strains, growth conditions, and plasmids.

Strains used in this study are listed in Table 1. C. difficile strains were grown at 37°C in an anaerobic chamber (85% nitrogen, 10% hydrogen, 5% carbon dioxide, Coy Lab Products) in brain heart infusion broth (BD Life Sciences) supplemented with 0.5% yeast extract (BD Life Sciences) and 0.1% cysteine (Sigma-Aldrich) (BHIS) or in C. difficile minimal media (CDMM) as described previously (26, 27). Escherichia coli strains were grown in lysogeny broth (LB) or agar (LBA), supplemented with 50 μg/ml kanamycin when necessary. Bacillus subtilis strains were grown on LBA or in BHI broth supplemented with 5 μg/ml tetracycline or 2.5 μg/ml chloramphenicol (27). All antibiotics were purchased from Sigma-Aldrich.

**TABLE 1:** Bacterial strains and plasmids used in this study.

Bacterial strain or plasmid	Relevant feature or genotype	Reference
*Clostridioides difficile R20291*		(29)
*Clostridioides difficile zupT*::*CT*	Intron inserted into *zupT*	This study
*Bacillus subtilis* JH BS2	Carries Tn196	(28)
*Escherichia coli* DH5α		(30)
*Escherichia coli* MG1655	RecA+	(31)
pJS107	ClosTron plasmid	(28)
pJS107_*zupT*	ClosTron plasmid with intron targeted to *zupT*	This study
pJS116	Stable *C. difficile* plasmid	(28)
pJS116_*pzupT-zupT*	*zupT*::*CT* complementation plasmid	This study

***zupT*::*CT* strain generation.** Gene inactivation was achieved using the ClosTron system as described previously (20, 27). Briefly, gBlocks containing specific modifications for insertion into the genome were generated using the TargeTronics algorithm (http://www.targetrons.com) and synthesized by Integrated DNA Technologies. The gBlocks were cloned into the pCR-Blunt vector using the Zero Blunt PCR cloning kit (Thermo Fisher Scientific) followed by restriction digest with BsrGI and HindIII (NEB) and ligation (NEB T4 ligase) into pJS107. Plasmids were transformed into the *recA*^+^ E. coli MG1655 through a standard heat shock protocol followed by transformation into B. subtilis JH2 using an established method (28). B. subtilis strains containing the pJS107_*zupT* plasmids were mated with C. difficile R20291 overnight at 37°C by plating and mixing together 100 μl of each strain onto a BHIS plate in the anaerobic chamber. Plates were scraped and transferred into 2 ml of BHIS prior to plating 200 μl onto BHIS plates containing 20 μg/ml thiamphenicol and 50 μg/ml kanamycin (BHIS_thiamp20kan50_). Colonies from these plates were patched onto new BHIS_thiamp20kan50_ and BHIS plates containing 5 μg/ml tetracycline (BHIS_tet5_). Patched colonies that were tetracycline sensitive were patched again onto new BHIS_thiamp20kan50_ and BHIS_tet5_ plates. Colonies that remained tetracycline sensitive were streaked onto BHIS plates containing 20 μg/ml lincomycin (BHIS_linc20_). Inactivation of the *zupT* gene was confirmed by performing PCR to identify a 1.5-kbp shift in gene size using gDNA extracted as previously described from colonies that were lincomycin resistant (27, 28).

**Complementation plasmids.** Complementation plasmids (Table 1) were created by amplifying the *zupT* gene and intergenic region from C. difficile strain R20291. The amplicon was then cloned into the pJS116 plasmid backbone. C. difficile strains were transformed as described above with the removal of the lincomycin selection and were maintained on BHIS_thiamp20_ to ensure plasmid retention (28).

**Growth assays.** Calprotectin was produced as described previously (6–9). Freshly streaked bacterial colonies were used to inoculate 5 ml of BHIS or BHIS_thiamp20_ and grown for 16 h at 37°C. Cultures were subcultured 1:50 into fresh BHIS or BHIS_thiamp20_ and grown for 6 h at 37°C prior to 1:50 inoculation into a 1:1 mixture of calprotectin buffer (20 mM Tris-HCl, 100 mM NaCl, 5 mM β-mercaptoethanol, 3 mM CaCl_2_) with BHIS or BHIS_thiamp20_ containing calprotectin or into BHIS or BHIS_thiamp20_ containing N,N,N’,N’,tetrakis (2-pyridinylmethyl)-1,2-ethanediamine (TPEN, Sigma) at the indicated concentrations. All growth assays were performed in a 96-well plate in 200 μl of media. Optical density at 600 nm (OD_600_) served as a measurement of growth and was measured every 30 min for the indicated total time in an EpochII microplate reader (BioTek).

### RNA extraction and quantitative qPCR.

C. difficile cultures were grown anaerobically in triplicate in a 1:1 mixture of calprotectin buffer and BHIS with calprotectin or TPEN added in indicated concentrations at 37°C to an OD_600_ of 0.3. After growth, a 1:1 solution of acetone:ethanol was added to an equal volume of the culture. Samples were stored at –80°C until used for RNA extraction. Samples were thawed on ice, pelleted, and resuspended in 750 μl of LETS buffer (1 M LiCl, 0.5 M EDTA, 1 M Tris pH 7.4). Cells were transferred to tubes containing lysing matrix B beads (MP Biomedicals) and lysed by a FastPrep-24 (MP Biomedicals) bead beater for 45 s at 6 m/s. Lysed samples were heated for 5 min at 55°C and pelleted by centrifugation for 10 min. The supernatant was transferred to a fresh tube and 1 ml TRIzol (Thermo Fisher Scientific) was added. Chloroform (200 μl) was added to each sample and vortexed prior to separation of the aqueous and organic layers by centrifugation for 15 min. The aqueous (upper) layer was transferred to a fresh tube and the RNA was precipitated through the addition of 1 ml isopropyl alcohol. Samples were incubated for 10 min and RNA was pelleted by centrifugation for 10 min. Supernatant was removed and the RNA pellet was washed with 200 μl of 70% ethanol. Samples were air dried for 1 min, then resuspended in 100 μl RNase free water. DNA contamination was removed through the addition of 8 μl RQ1 DNase, 12 μl 10× RQ1 buffer, and 2 μl RNase inhibitor (Promega) to the purified RNA. Samples were DNase treated for 2 h and purified using the RNeasy miniprep RNA cleanup kit (Qiagen). RNA concentration was determined using the Synergy 2 with Gen 5 software (BioTek) and 2 μg was reverse transcribed by M-MLV reverse transcriptase (Thermo Fisher Scientific) in the presence of RNase inhibitor (Promega) and random hexamers (Promega). Reaction mixtures lacking the reverse transcriptase were used to control for DNA contamination. Newly created cDNA was diluted 1:100 and was used in qRT-PCR using iQ SYBR green supermix (BIO-RAD) utilizing the primer pairs in Table 2. Amplification was achieved using a 3-step melt cure program on a CFX96 qPCR cycler (BIO-RAD). Transcript abundance was calculated using the ΔΔCT method normalized by the *rpoB* gene.

**TABLE 2:** Oligonucleotides used in this study

Name	Sequence (5′ to 3′)	Description
qRT_*zupT*_F	ccagtttattatgccacaggag	qRT-PCR forward primer for *zupT*
qRT_*zupT*_R	tcgctcctaaaggctcagac	qRT-PCR reverse primer for *zupT*
qRT_*rpoB*_F	tgctgttgaaatggttcctg	qRT-PCR housekeeping gene forward primer
qRT_*rpoB*_R	cggttggcatcatcattttc	qRT-PCR housekeeping gene reverse primer
R20291_*zupT* _F	agcttaggattttctgctggtgt	Forward primer to check for intron insertion into *zupT*
R20291_*zupT*_R	tcgctcctaaaggctcagac	Reverse primer to check for intron insertion into *zupT*

### Animals and experimental models of Clostridioides difficile infection.

All animal experiments were approved by the Animal Care and Use Committee of Vanderbilt University (protocol M1700053). C57BL/6 male mice at 6 to 8 weeks old were purchased from Jackson Laboratories and given one week to equilibrate their microbiota prior to experimentation. Mice were housed with autoclaved water, food, and bedding and all experimental manipulations were performed in a biosafety level 2 laminar flow hood. Mice were housed in individual cages under the same conditions during the experiment and all mice were culture negative for C. difficile prior to infection. One week prior to infection, mice were placed on diets containing altered metal levels as previously described (7). For the C. difficile infection model, mice were given cefoperazone at 0.5 mg/ml in drinking water *ad libitum* for 5 days followed by a 2-day recovery period and subsequent infection. Mice were infected via oral gavage with a 1:1 mixture of 1 × 10^5^ spores of C. difficile (NAP1/BI/027 strain R20291) and *zupT*::*CT* or 1 × 10^5^ spores of each individual strain resuspended in phosphate-buffered saline (PBS). Mice were monitored for survival or were euthanized after reaching a terminal endpoint of appearing moribund or experiencing weight loss of >20% from baseline. C. difficile CFU were quantified daily from fecal samples. Samples were diluted and homogenized in PBS and serial plated onto taurocholate cycloserine cefoxitin fructose agar (TCCFA) with or without lincomycin for enumeration as CFU per gram of feces. Competitive index (*zupT*::CT/WT) was measured for each dietary condition.

### Uptake of ^70^Zn isotope.

C. difficile strains were grown anaerobically in BHIS or BHIS_thiamp20_ containing 50 μM TPEN to an OD_600_ of 0.5. ^70^ZnO (Cambridge Isotope Laboratories) was added to each sample at a final concentration of 25 μM and samples were incubated at 37°C for 1 h. A 1:1 mixture of acetone:ethanol was added and the cells were pelleted by centrifugation (4000 × *g* for 10 min). Cells were subsequently washed twice and resuspended in 1× PBS. Samples were transferred to metal-free 15 ml conical tubes (VWR), digested overnight in 50% Optima-grade nitric acid (Thermo Fisher Scientific) and 12.5% hydrogen peroxide at 50°C prior to 1:10 dilution in Millipore water for inductively coupled plasma mass spectrometry analysis.

### Inductively coupled plasma mass spectrometry.

Elemental quantification on acid-digested liquid samples was performed using an Agilent 7700 inductively coupled plasma mass spectrometer (Agilent, Santa Clara, CA) attached to a Teledyne CETAC Technologies ASX-560 autosampler (Teledyne CETAC Technologies, Omaha, NE). The following settings were fixed for the analysis: Cell Entrance = −40 V; Cell Exit = −60 V; Plate Bias = −60 V; OctP Bias = −18 V; and collision cell Helium Flow = 4.5 ml/min. Optimal voltages for Extract 2, Omega Bias, Omega Lens, OctP RF, and Deflect were determined empirically before each sample set was analyzed. Element calibration curves were generated using ARISTAR ICP Standard Mix (VWR, Radnor, PA). Samples were introduced by peristaltic pump with 0.5 mm internal diameter tubing through a MicroMist borosilicate glass nebulizer (Agilent). Samples were initially taken up at 0.5 rps for 30 s followed by 30 s at 0.1 rps to stabilize the signal. Samples were analyzed in Spectrum mode at 0.1 rps, collecting three points across each peak and performing three replicates of 100 sweeps for each element analyzed. The sampling probe and tubing were rinsed for 20 s at 0.5 rps with 2% nitric acid between every sample. Data were acquired and analyzed using the Agilent Mass Hunter Workstation Software version A.01.02. Data of the investigated metal ion were normalized to the ^34^S abundance of each sample.

### Statistical analyses.

Statistical analyses were performed using GraphPad Prism version 8. Specific statistical tests, replicate numbers, calculated errors, and other information for each experiment are reported in the figure legends.
